# The DIVIPACT cohort profile: Evaluating the impact of colonic diverticulosis on daily life

**DOI:** 10.1111/codi.70155

**Published:** 2025-06-27

**Authors:** Helene R. Dalby, Rune Erichsen, Kåre A. Gotschalck, Katrine J. Emmertsen

**Affiliations:** ^1^ Department of Surgery Randers Regional Hospital Randers Denmark; ^2^ Department of Surgery Horsens Regional Hospital Horsens Denmark; ^3^ Department of Clinical Medicine Aarhus University Aarhus Denmark

**Keywords:** cohort study, diverticular disease, diverticulosis, DIVIPACT, PROM, quality of life

## Abstract

**Aim:**

Colonic diverticulosis is asymptomatic in most subjects but can impact daily life. The DIVIPACT study comprehensively explores the impact of diverticulosis on daily life. The aim of this publication is to introduce the DIVIPACT cohort, present self‐reported data from a cross‐sectional survey and outline the potential for further research.

**Method:**

The DIVIPACT cohort comprises subjects diagnosed with diverticulosis (K572–9) in the Central Denmark Region (five hospitals, ~1.3 million residents) between 2010 and 2022 who responded to an online questionnaire survey conducted in 2023 assessing health factors and quality of life (QoL). Self‐reported data were linked to Danish health registries for consenting responders. Responders were categorized according to previous hospital management (inpatient, outpatient or diverticulosis) and characterized based on self‐reported health factors.

**Results:**

Of the 20 961 responders (74% response rate), 19 244 (92%) consented to data linkage. Among these, 4184 (22%) were inpatients, 8666 (45%) were outpatients and 6394 (33%) had diverticulosis only. Overall, 1596 (10%) reported restrictions on activity due to diverticulosis in the past 4 weeks. Bowel function affected QoL in 66% of inpatients, 54% of outpatients and 44% of diverticulosis‐only individuals.

**Conclusion:**

The DIVIPACT cohort provides one of the most extensive datasets available for evaluating the impact of colonic diverticulosis on daily life, addressing important knowledge gaps and providing a foundation for patient‐centred management strategies.

**What does this study add to the literature?**

This study addresses critical gaps in understanding the burden of diverticulosis by establishing the DIVIPACT cohort, comprising 20 961 respondents (74% response rate) to a comprehensive survey. It provides one of the most extensive datasets linking self‐reported data with Danish registries, enabling detailed analyses of the impact of diverticulosis on daily life.

## INTRODUCTION

Colonic diverticulosis is an asymptomatic anatomical abnormality representing the most frequently observed finding during routine colonoscopy, with prevalence rates ranging from 10% in individuals under 40 years to 70% in those over 80 years [1, 2]. While most individuals with diverticulosis remain asymptomatic, symptomatic diverticular disease (DD) is among the leading causes of hospitalization for digestive disorders, with a lifetime risk from age 30 at 3% in men and 5% in women [1, 3].

The impact of diverticulosis on quality of life (QoL) is increasingly acknowledged [2, 4, 5, 6, 7, 8]. QoL is a multidimensional concept comprising physical, psychological, mental and social aspects, all of which may be impacted by diverticulosis. Current guidelines for diverticulosis acknowledge QoL as an essential consideration alongside clinical outcomes in diverticulosis management [9, 10]. However, most studies investigating QoL with diverticulosis are based on patients with admissions for DD [8, 11, 12, 13, 14, 15, 16, 17], thus limiting generalizability to the broader population with diverticulosis. As a result, critical knowledge about the everyday burden of diverticulosis, particularly in subjects with no admissions, is lacking, and the psychosocial and functional dimensions critical to understanding the burden of diverticulosis are less well characterized. Addressing this gap is essential for developing more holistic, patient‐centred management strategies.

By using a comprehensive questionnaire survey within a population‐based cohort, the DIVIPACT study seeks to provide a more comprehensive understanding of the impact of diverticulosis on daily life. This publication introduces the DIVIPACT cohort, designed to examine the impact of diverticulosis on daily life across a representative population. We describe how the cohort was assembled, present key patient‐reported data and outline the potential for further research utilizing this cohort.

## METHOD

This study is reported following the Strengthening in Reporting of Observational Studies in Epidemiology (STROBE) Statement guidelines for reporting cross‐sectional studies [18].

### Design

**FIGURE 1:**
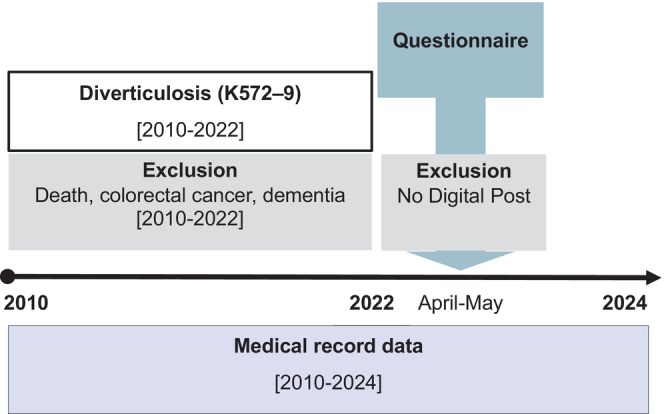
Study design.

The DIVIPACT cohort comprises respondents to a cross‐sectional online questionnaire survey conducted in Denmark in April–May 2023 (Figure 1).

### Setting

Denmark has a universal tax‐supported public healthcare system that provides free healthcare to all residents. National healthcare has been managed through five regions since 2007 [19]. The Central Denmark Region (five public hospitals, ~1.3 million residents) has used electronic health records since 2010. Data from the electronic health records are stored in the Business Intelligence (BI) Data Warehouse, which integrates data collected from, for example, health records, laboratory, radiology, pharmacy and administrative systems [20]. All hospital contacts are recorded with one primary diagnosis and potentially multiple secondary diagnoses coded using the International Classification of Diseases, Tenth Revision (ICD‐10). Data entries in the BI Data Warehouse align with national registries known to have high validity and completeness [21, 22]. The unique personal civil registration (CPR) number assigned to all residents in Denmark allows unambiguous linkage across the registries at an individual level.

### Study cohort

Subjects were identified via the BI Data Warehouse in August 2022. Eligible subjects included all with a diagnosis of diverticulosis (ICD‐10 codes K57.2–9) as either a primary or secondary diagnosis at an inpatient or outpatient contact to any public hospital in the Central Denmark Region from 2010–2022.

Those diagnosed with colorectal cancer or dementia, deceased subjects and subjects unable to receive digital mail from public Danish authorities were excluded.

### The questionnaire

#### Developing the questionnaire

We developed a comprehensive online questionnaire designed for completion on any type of electronic device. The main topics covered included experience with diverticulosis and self‐assessed health factors. In addition to several ad hoc questions, validated patient‐reported outcome measures (PROMs) were incorporated to assess QoL (Table S1).

#### Patient‐reported outcome measures

Disease‐specific QoL was assessed by the diverticulitis QoL (DV‐QOL) instrument [5], while generic QoL was assessed by the EuroQol 5 Dimension 5 Level survey (EQ‐5D‐5L) and visual analogue scale (VAS) [23]. The colostomy impact score evaluated the impact of colostomy dysfunction on QoL [24]. To assess the impact of chronic pain on QoL, we used a modified version of the scoring system developed to evaluate chronic pain after rectal cancer surgery [25]. The impact of lower urinary tract symptoms on QoL was evaluated by the International Consultation on Incontinence Questionnaire for women (ICIQ‐FLUTS) and men (ICIQ‐MLUTS), respectively [26, 27]. The Rectal Cancer Female Sexuality Score evaluated the impact of sexual function on QoL in women [28]; for men we used the International Index of Erectile Function (IIEF) Questionnaire [29].

#### Pilot test of the questionnaire

The questionnaire was piloted to ensure comprehensibility by sending it to 100 subjects in the target population. They provided comprehensive written and oral feedback, and the questionnaire was revised according to their comments and suggested revisions.

### Data collection

All Danish residents aged ≥15 years with a CPR number are required to receive digital mail from public authorities unless exempt [30]. Since its introduction by the Danish government in 2014, the secure digital mailing system e‐Boks has registered 5.2 million users, representing 88% of the population [30, 31, 32].

Eligible subjects received an invitation letter in e‐Boks, distributed through the online platform SurveyXact [33]. The secure mailing system ensured that only the intended subjects could open the invitation letter. The invitation letter included a link to the online questionnaire and links to webpages with more information about the study and data security [34, 35]. Contact information for the primary investigator (HD) was included in both the invitation letter and the webpages, enabling subjects to address any questions or concerns via telephone or email. Subjects were informed that participation was voluntary and that their responses were anonymized.

Nonresponders received reminders on days 7, 14, 30 and 60. The questionnaire was distributed in eight rounds during April and May 2023, with all questionnaires closed for response by September 2023.

#### Record linkage to the Danish Health Registries

Using CPR numbers, individual responders could be linked to information in the BI Data Warehouse. After being thoroughly informed, participants were asked to consent to data linkage. The questionnaire's completion was independent of whether participants provided consent for data linkage. Data extraction for participants who consented was conducted in February 2024. Extracted information included detailed records of hospital contacts and surgical procedures, descriptions of CT scans and results from laboratory and pathological examinations.

#### Target population characterization

Extraction of sex and age from the CPR number was applied for all eligible subjects.

### Cohort characteristics

Responders were characterized according to self‐reported health factors. Height and weight were used to calculate body mass index (BMI), which was classified according to the WHO as underweight, normal weight, overweight or obese [36]. Additional health factors included smoking status, alcohol consumption, moderate and vigorous physical activity, self‐rated performance status and comorbidities. Responders consenting to data linkage were classified according to the level of hospital management for diverticulosis based on information obtained from the BI data as: (1) those with previous *inpatient* contact with DD as the primary diagnosis, (2) those with *outpatient* contact with DD as the primary diagnosis and (3) those with *diverticulosis*, i.e. diverticulosis recorded solely as a secondary diagnosis in any hospital contact. Disease severity was extracted and categorized as complicated if a diagnosis of abscess, perforation, fistula or stricture was registered, and otherwise as uncomplicated.

### Impact of diverticulosis on daily life

In this paper we report the results of some ad hoc questions to categorize the cohort, including foods affecting bowel function, treatment for diverticulosis [medication to regulate bowel function, contact with general practitioner (GP), antibiotics, and surgery], knowledge of the condition and perceived level of counselling for diverticulosis. Additionally, we characterize QoL by three questions concerning restrictions on daily activities caused by diverticulosis, the effect of bowel function on QoL and overall QoL.

### Statistical analysis

Descriptive statistics were compiled to characterize the responders consenting to data linkage according to their self‐reported health factors. Quantitative data were estimated as median with interquartile range (IQR) and categorical data as absolute numbers and percentages.

Pearson's chi‐square test or the Kruskal–Wallis rank sum test was applied as appropriate to test for statistical differences between the groups. The correlation between days feeling restricted because of diverticulosis and QoL was investigated by visualizing the fit between the responses.

#### Missing data

Proportions for questionnaire items were calculated based on item‐specific response rates to accommodate partial responses. Statistical analyses were conducted using RStudio, version 2024.04.2+764 (Posit PBC).

## RESULTS

### Target population characteristics

**FIGURE 2:**
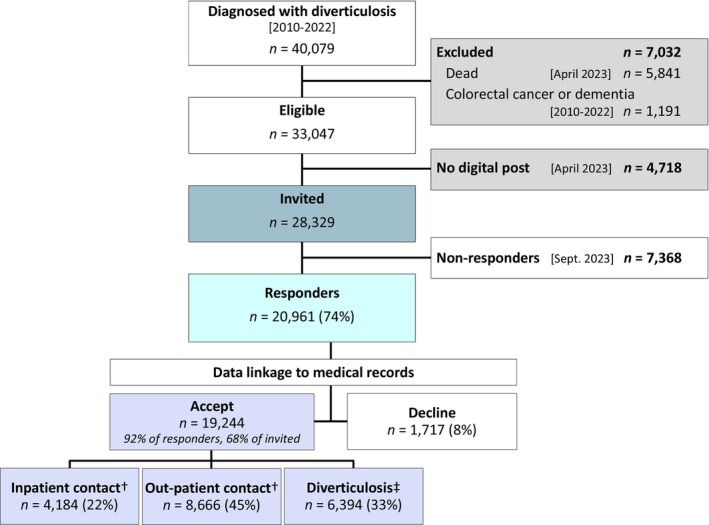
Patient flow in the study. ^†^Diverticular disease as the primary diagnosis. ^‡^No hospital contact with diverticular disease as the primary diagnosis.

The target population comprised 40 079 subjects diagnosed with diverticulosis, of whom 33 047 (82%) were eligible for inclusion. A total of 28 329 subjects were able to receive digital mail and therefore were invited to participate (Figure 2). The target population had a median age of 74 (IQR 64–81) years, with 53% being female, while eligible subjects had a median age of 71 (IQR 63–78) years (53% female). Subjects without digital mail were older than those invited to participate [median age 82 (IQR 75–87) versus 70 (IQR 61–77) years], and women constituted 63% versus 52% of those invited. Of the invited subjects, 20 961 (74%) responded. Responders had a median age of 70 (IQR 63–77) years when answering the questionnaire, and women constituted 52%. All nonparticipants, i.e. all subjects who were excluded, not invited or did not respond, were older, with a median age of 78 (IQR 68–86) than responders, while the sex distribution was consistent with 53% being female.

### Cohort characteristics

Most responders went through the entire questionnaire (*n* = 16 784, 88%) and spent a median of 18 (IQR 14–26) min on their response. Almost all (*n* = 19 244, 92%) responders consented to data linkage. Responders who consented to or declined data linkage had the same median age, but women constituted 62% of those who declined and 52% of those who accepted. All other self‐reported health factors were similar between those who consented to or declined data linkage.

Of those consenting to data linkage, 4184 (22%) had a previous *inpatient* contact with DD as the primary diagnosis, 8666 (45%) had *outpatient* contact with DD as the primary diagnosis and 6394 (33%) responders were classified as having *diverticulosis*. The median time from diagnosis of diverticulosis to survey completion was 55 months (IQR 29–88), with the longest interval being observed in those with an inpatient contact and the shortest in those classified as having diverticulosis (Table 1).

**TABLE 1:** Characteristics of subjects in the DIVIPACT cohort.

Characteristic	*N*	Inpatient (*n* = 4184; 22%)	Outpatient (*n* = 8666; 45%)	Diverticulosis (*n* = 6394; 33%)
Sex	19 244			
Female		2600 (62)	4477 (52)	2845 (44)
Male		1584 (38)	4189 (48)	3549 (56)
Age (years)	19 244	66 (57–74)	71 (64–77)	72 (65–77)
Age group (years)	19 244			
<60		1244 (30)	1221 (14)	704 (11)
60–69		1291 (31)	2317 (27)	1820 (28)
70 or older		1649 (39)	5128 (59)	3870 (61)
Time from diagnosis of diverticulosis to survey (months)	19 244	67 (39–100)	57 (30–91)	46 (25–73)
Disease severity	19 244			
Uncomplicated		3424 (82)	8609 (99)	6375 (100)
Complicated^a^		760 (18)	57 (0.7)	19 (0.3)
Number of hospital contacts with diverticulosis as primary diagnosis	19 244			
0		0	0	6394 (100)
1		982 (23)	7153 (82)	0
2–3		2147 (51)	1400 (16)	0
4 or more		1055 (25)	113 (1)	0
Self‐reported health factors				
BMI (kg/m^2^)	16 868			
Underweight (<18.5)		21 (1)	85 (1)	48 (1)
Normal (18.5–24.9)		1073 (29)	2641 (35)	1739 (31)
Overweight (25–29.9)		1479 (40)	3047 (40)	2335 (42)
Obese (30+)		1156 (31)	1806 (24)	1438 (26)
Smoking status	17 260			
Never		1537 (40)	3191 (41)	2169 (38)
Former		1837 (48)	3786 (49)	2858 (50)
Active smoker		431 (11)	789 (10)	662 (12)
Alcohol (units/week)	17 167			
<10		3306 (87)	6437 (83)	4532 (80)
>10		482 (13)	1291 (17)	1119 (20)
Physical activity (time/week)	16 611			
Less than 15 min		664 (18)	1201 (16)	877 (16)
15 min to 2 h		1642 (45)	3388 (45)	2476 (45)
2 h or more		1329 (37)	2885 (39)	2149 (39)
Self‐rated performance status	16 964			
Fully active		2057 (55)	4198 (55)	3036 (54)
Restricted, but ambulatory		1407 (38)	2876 (38)	2155 (38)
Ambulatory, but no hard work		186 (5)	364 (5)	290 (5)
Limited self‐care or completely disabled		93 (2)	175 (2)	127 (2)
Comorbidities				
No known diseases	18 906	1075 (26)	2048 (24)	1511 (24)
Hypertension	18 906	1518 (37)	3489 (41)	2698 (43)
Chronic pain	18 906	795 (19)	1427 (17)	938 (15)
Cured of a previous cancer	18 840	396 (10)	967 (11)	838 (13)
Diabetes	18 906	381 (9)	864 (10)	709 (11)
COPD	18 906	229 (6)	591 (7)	516 (8)
Asthma	18 906	380 (9)	684 (8)	485 (8)
Depression and/or anxiety	18 840	404 (10)	677 (8)	452 (7)
Cancer	18 840	109 (3)	258 (3)	175 (3)

*Note*: Data presented as *n* (%) or median (IQR).

Abbreviations: BMI, body mass index; COPD, chronic obstructive pulmonary disease.

^a^ Complicated disease includes abscess, perforation, fistula and stricture.

### Self‐reported health factors

The median BMI was 26.8 (IQR 24.2–30.1) kg/m^2^ and 67% (*n* = 11 261) were overweight or obese with a BMI ≥ 25 kg/m^2^. Based on self‐rated performance status, 9291 (55%) were fully active. Among reported comorbidities, hypertension was the most common (41%, *n* = 7705), but chronic pain, previous cancer and diabetes were each reported by at least 10% of respondents (Table 1).

### Impact of diverticulosis on daily life

#### Food affecting bowel function

**FIGURE 3:**
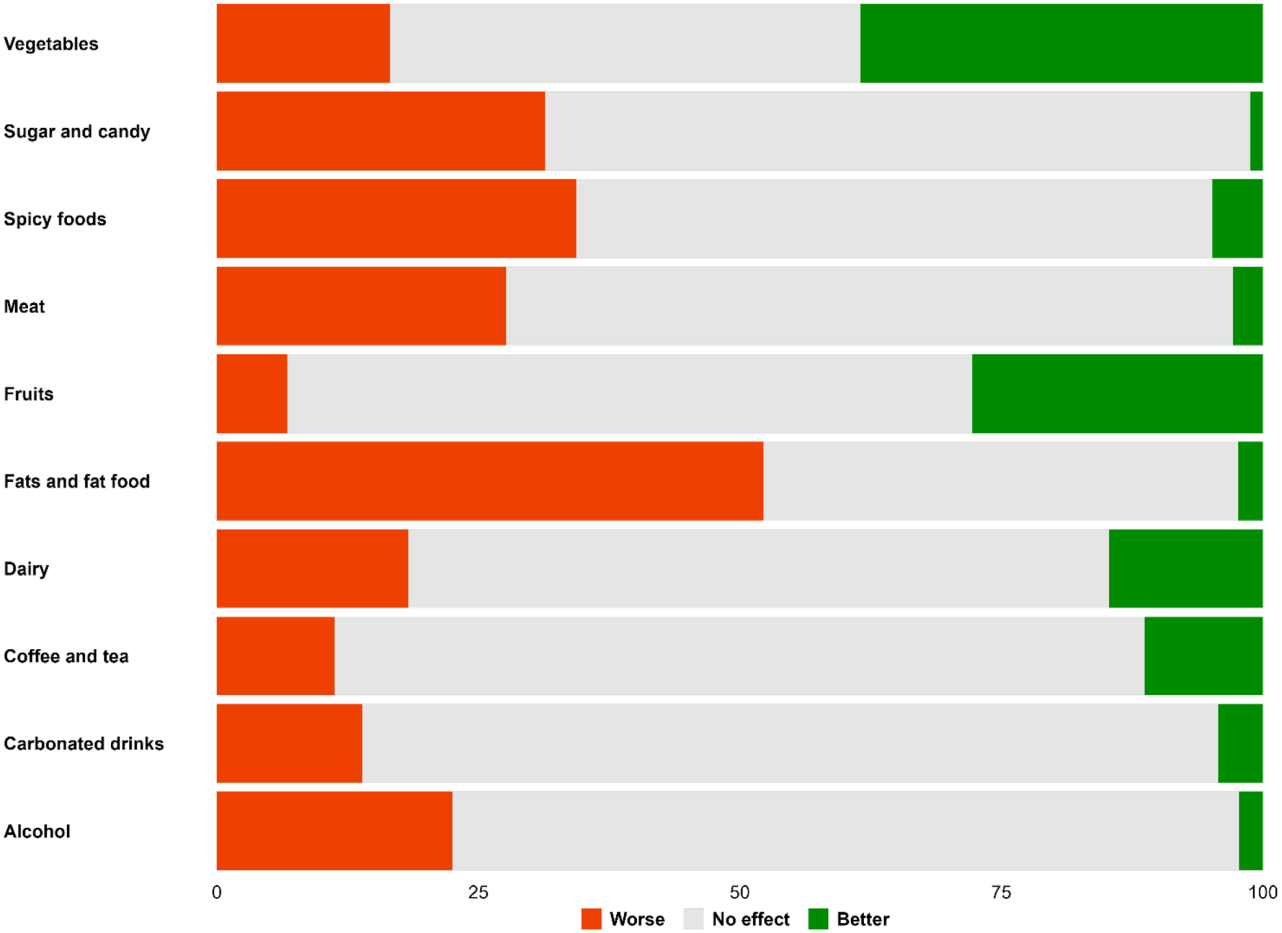
Specific food categories affecting bowel function reported by 11 741 of the responders when asked if the specific food category made their bowel function ‘Worse’, ‘Better’ or had ‘No effect’.

Subjects responding ‘Yes’ (*n* = 5744) or ‘Don't know’ (*n* = 5997) to the question ‘Does specific food affect your bowel function?’ were asked to indicate if ten specific food categories made bowel function better or worse or had no effect (Figure 3). Vegetables and fruits were reported to make bowel function better, while fats, spicy foods, sugar and candy, and meat were reported to make bowel function worse. Responders with previous inpatient or outpatient contact more often reported that specific foods affected their bowel function than responders with diverticulosis (Table 2).

**TABLE 2:** Self‐reported impact of diverticulosis on daily life for subjects in the DIVIPACT cohort.

Question	*N*	Inpatient (*n* = 4184; 22%)	Outpatient (*n* = 8666; 45%)	Diverticulosis (*n* = 6394; 33%)
Symptoms:				
Do specific foods affect your bowel function?	16 920			
Don't know		1163 (32)	2771 (36)	2063 (37)
No		826 (22)	2317 (30)	2036 (36)
Yes		1699 (46)	2531 (33)	1514 (27)
Have you experienced rectal bleeding?	19 244			
No bleeding		3117 (74)	6174 (71)	4648 (73)
Yes, but no contact with a medical doctor		637 (15)	1423 (16)	1067 (17)
Yes, with contact with a medical doctor		430 (10)	1069 (12)	679 (11)
*Treatment*				
Do you use bulking agents?	16 709			
No		1773 (48)	4288 (57)	3538 (64)
Yes, sometimes		624 (17)	1028 (14)	660 (12)
Yes, every day		1309 (35)	2178 (29)	1311 (24)
Do you use laxatives?	16 382			
No		2455 (66)	5612 (75)	4361 (79)
Yes, sometimes		523 (14)	873 (12)	576 (10)
Yes, every day		732 (20)	999 (13)	577 (10)
Do you use antidiarrhoeals?	16 708			
No		3322 (91)	6700 (91)	5002 (92)
Yes, sometimes		235 (6)	436 (6)	300 (6)
Yes, every day		90 (3)	188 (3)	109 (2)
Have you contacted your general practitioner due to diverticular disease?	16 439			
Don't know		263 (7)	409 (6)	266 (5)
No		2000 (54)	6107 (83)	4788 (89)
Yes		1408 (38)	866 (12)	332 (6)
Did your general practitioner prescribe you antibiotics due to diverticular disease?	16 392			
Don't know		183 (5)	232 (3)	154 (3)
No		2357 (64)	6462 (88)	4955 (92)
Yes		1122 (31)	669 (9)	258 (5)
Have you had surgery due to diverticular disease?	17 227			
No		3091 (81)	7319 (95)	5167 (91)
Yes		673 (19)	405 (5)	542 (9)
Do you have an intestinal stoma?	19 244			
No		3981 (95)	8617 (99)	6355 (99)
No, but I had a stoma that has been reversed		94 (2)	32 (<1)	22 (<1)
Yes		109 (3)	17 (<1)	17 (<1)
Knowledge and counselling:				
Did you know you have diverticulosis?	17 002			
Yes		2950 (79)	4868 (64)	2965 (52)
No		763 (21)	2770 (36)	2686 (48)
How do you perceive the availability of counselling with regard to diverticulosis?	16 467			
Comprehensive		375 (10)	522 (7)	292 (5)
Acceptable		639 (18)	756 (10)	435 (8)
Deficient		663 (18)	959 (13)	544 (10)
Not sought		1458 (41)	4200 (57)	3415 (63)
Don't know		457 (13)	975 (13)	777 (14)
Quality of life:				
How many days during the past 4 weeks have diverticular disease restricted you from carrying out your daily activities?	16 450			
None		3067 (84)	6736 (91)	5051 (94)
1–2 days		318 (9)	372 (5)	171 (3)
3–7 days		163 (4)	173 (2)	95 (2)
8–14 days		54 (2)	41 (1)	26 (1)
14+ days		69 (2)	72 (1)	42 (1)
Overall, how much does your bowel function affect your quality of life?	16 587			
Not at all		1263 (34)	3439 (46)	3049 (56)
A little		1470 (40)	2679 (36)	1696 (31)
Quite a bit		656 (18)	984 (13)	550 (10)
Extremely		282 (8)	337 (5)	182 (3)
Overall, how do you rate your quality of life over the past 4 weeks?	16 585			
Very bad		46 (1)	65 (1)	37 (1)
Bad		459 (13)	682 (9)	433 (8)
Good		2044 (56)	3996 (54)	2756 (50)
Very good		1121 (31)	2696 (36)	2250 (41)

*Note*: Data presented as *n* (%).

### Treatment for diverticulosis

#### Medication to regulate bowel function

Overall, 7110 (43%) responders reported using bulking agents and 4280 (26%) reported using laxatives, both more frequently reported by responders with inpatient and outpatient contacts than those with diverticulosis (Table 2).

#### General practitioner

Consultations with their GP due to DD were reported by 2606 (16%) responders, of whom 2049 (79%) reported they had been treated with antibiotics prescribed by their GP. A notably higher proportion of responders with previous inpatient (38%) or outpatient (12%) contact had contacted their GP than those with diverticulosis (6%) (Table 2).

#### Surgery

A total of 1650 (10%) reported having undergone surgery due to DD (Table 2), while 143 (1% of all responders and 9% of responders reporting having undergone surgery) had a stoma at the time of response. An additional 148 responders reported having had a previous stoma reversed.

### Knowledge and counselling

A total of 6219 (37%) respondents reported being unaware of having diverticulosis. Notably, more of those with diverticulosis (48%) than those with outpatient (36%) or inpatient (21%) contacts reported being unaware of their condition.

Regarding the perception of counselling for diverticulosis, only 3019 (18%) perceived the level of counselling to be comprehensive or acceptable. A remarkably higher proportion of those with inpatient (18%) or outpatient (13%) contact than those with diverticulosis (10%) perceived the availability of counselling to be deficient (Table 2).

### Quality of life

#### Daily activities restricted by diverticula

A total of 1596 (10%) people reported that DD had restricted them from carrying out their daily activities on at least 1 day during the past 4 weeks, mostly for those with previous inpatient (16%) or outpatient (9%) hospital contact than those with diverticulosis (6%) (Table 2). Responders reporting that they had undergone surgery due to DD were more likely to report that DD had restricted them from carrying out their daily activities compared with those reporting no surgery (13% vs. 9%), which was also true for those with complicated disease compared with those with uncomplicated disease (16% vs. 9%). Accordingly, the proportion of patients reporting restrictions on activity increased with increasing hospital contacts from 6% of those with no hospital contacts, 8% of those with one hospital contact, 14% of those with two or three hospital contacts and 14% of those with four or more hospital contacts. All differences were statistically significant.

#### Bowel function affecting quality of life

Most responders with previous inpatient or outpatient contact reported that their bowel function affected their QoL (66% and 54%, respectively), but this was also reported by a significant proportion of those with diverticulosis (44%) (Table 2). Responders reporting that they had undergone surgery due to DD were more likely to report that their bowel function affected their QoL than those reporting no surgery (60% vs. 53%). The same was found in responders with complicated disease versus uncomplicated disease (67% vs. 53%). Accordingly, the proportion of patients reporting that their bowel function affected their QoL increased with increasing hospital contacts from 44% of those with no hospital contacts, 52% of those with one hospital contact, 63% of those with two or three hospital contacts and 78% of those with four or more hospital contacts. All differences were statistically significant.

#### Overall quality of life

Overall QoL during the past 4 weeks was evaluated to be good or very good by 90% of responders overall (Table 2). Responders reporting that they had undergone surgery due to DD were more likely to report that their overall QoL was bad or very bad than those reporting no surgery (14% vs. 10%). The same was found in responders with complicated disease versus uncomplicated disease (17% vs. 10%). Accordingly, the proportion of patients reporting their overall QoL to be bad or very bad increased with increasing hospital contacts from 8% of those with no hospital contacts, 10% of those with one hospital contact, 12% of those with two or three hospital contacts and 20% of those with four or more hospital contacts. All differences were statistically significant.

#### Correlation between days feeling restrictions because of diverticulosis and quality of life

**FIGURE 4:**
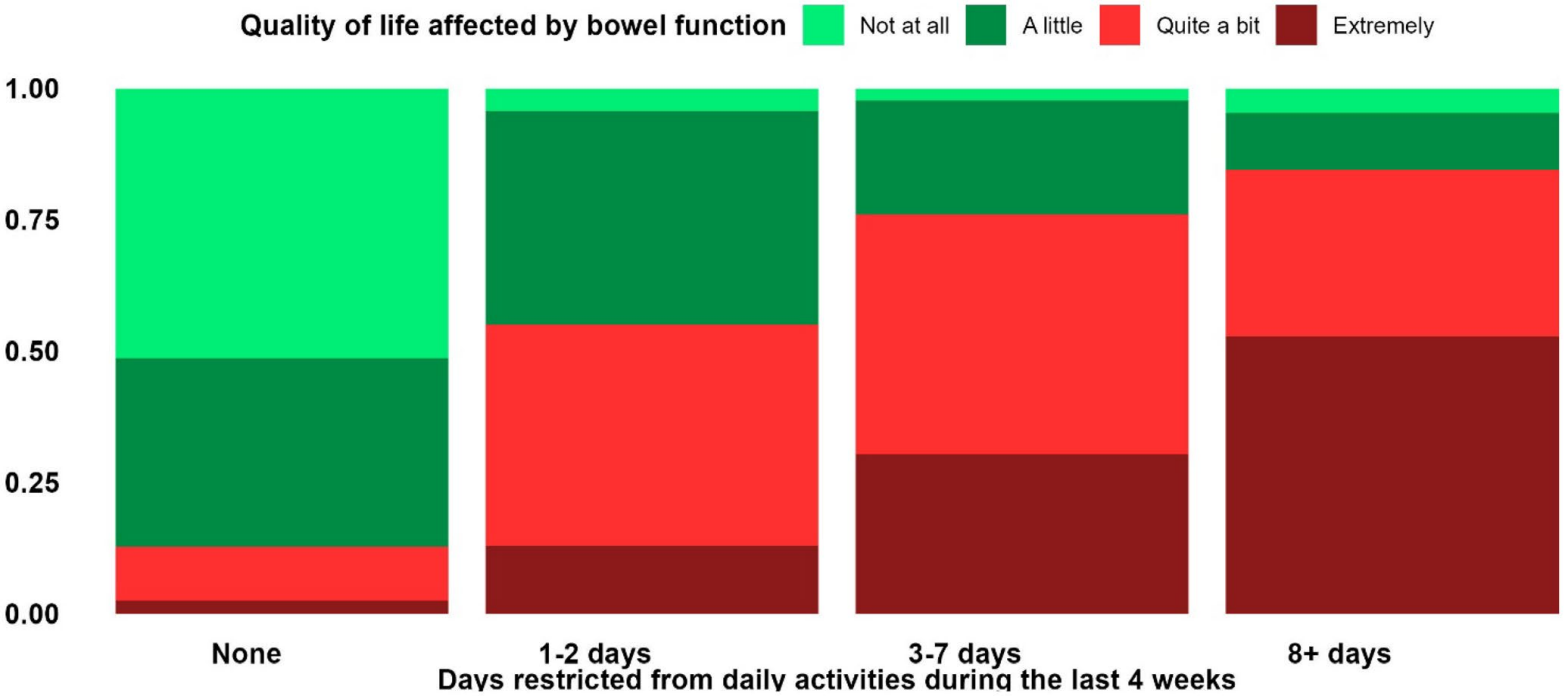
Correlation between reported restrictions from carrying out daily activities and the impact of bowel function on quality of life.

Responders reporting restrictions from daily activities because of diverticulosis accordingly reported that their bowel function affected their QoL and evaluated their QoL as worse than those reporting no restrictions. The correlation between days feeling restrictions because of diverticulosis and the impact of bowel function on QoL is visualized in Figure 4.

## DISCUSSION

The DIVIPACT cohort represents one of the largest and most detailed datasets available for evaluating the impact of colonic diverticulosis on daily life. By including individuals who have been typically underrepresented in previous studies on QoL in diverticulosis, this study fills a critical gap in understanding the broader implications of the condition. The use of a comprehensive questionnaire enables analysis of diverse aspects of daily life. Furthermore, the ability to link self‐reported data with the Danish Health Registries facilitates a detailed characterization of the clinical course and allows for the correlation of self‐reported outcomes with clinical metrics.

To ensure participant compliance while maximizing data quality, the questionnaire was designed to be both thorough and concise. Short, yet adequate, PROMs were preferred to minimize inconvenience for responders, with a preference for PROMs previously utilized in assessing QoL in patients with gastrointestinal disorders. The digital distribution of the questionnaire allowed us to invite thousands of subjects and increased convenience for responders. The high response rate of 74% underscores the feasibility of this approach and supports the cohort's external validity. Other studies investigating QoL in diverticulosis in cross‐sectional designs report response rates of 17%–53% [14, 37], and the largest cohort in previous studies examining QoL in diverticulosis investigated 1206 subjects [13].

The mean age of the DIVIPACT cohort is higher than in many QoL studies [13, 14, 15, 17], offering a valuable perspective on an older population that is frequently underrepresented in clinical trials. Importantly, as the prevalence of diverticulosis increases with age, the median age of 70 years in the DIVIPACT cohort is assumed to better represent the actual population with diverticulosis. Nonetheless, we have a subgroup of 3169 responders below the age of 60, which enables us to further investigate the impact of diverticulosis in younger subjects.

Most studies investigating the impact of diverticulosis on QoL are based on patients with admissions for acute diverticulitis [8, 11, 12, 13, 14, 15, 16, 17]. QoL in subjects with DD managed as outpatients only has been investigated in three studies, all evaluating the effect of pharmacological treatment (antibiotics and/or probiotics) [13, 38, 39]. These studies consistently reported improved QoL in pharmacologically treated subjects compared with untreated subjects [13, 38] or showed improvement in QoL from baseline to posttreatment [39].

Only one study has investigated subjects without hospital contact primarily due to diverticulosis [40]. That study evaluated constipation and QoL using the SF‐12 in 290 subjects with and 686 without diverticulosis and found no association between diverticulosis and constipation but a significantly reduced QoL in those with diverticulosis. Our study addresses critical gaps in generalizability by including subjects who have previously received limited attention. Additionally, it expands our understanding of the impact of diverticulosis by exploring a wider range of effects on daily life and leveraging the ability to link self‐reported outcomes with comprehensive health registry data.

In line with prior research, our findings confirm that individuals with diverticulosis experience a range of symptoms affecting QoL [2, 41]. The effects of diverticulosis on daily life were most significant for individuals with previous hospital stays, had a moderate impact on those with outpatient visits and were least severe for those with diverticulosis. Accordingly, responders who had undergone surgery or experienced complicated disease reported a significantly greater impact of diverticulosis on daily life compared with those without surgery or with uncomplicated disease. Furthermore, the impact on QoL increased with the number of hospital contacts. These findings highlight specific patient groups that require particular attention.

Despite the subgroup differences, nearly half of the participants with uncomplicated diverticulosis and those with no hospital contacts reported that their bowel function had a negative influence on their QoL, and 6% indicated that DD had restricted them from carrying out their daily activities. Understanding the impact of DD is essential for improving patient management. Given the high prevalence of diverticulosis and the substantial financial burden of hospitalizations [42, 43, 44], optimizing patient management has significant implications for patients, primary and secondary healthcare systems, and society.

The questionnaire included ad hoc questions and PROMs assessing functional outcomes and health‐related QoL. However, except for the DV‐QOL, none of the applied PROMs have been validated for subjects with diverticulosis. Previous studies have assessed generic QoL using the EQ‐5D‐5L [45, 46]. The other PROMs were originally designed for men with prostatic disease (MLUTS [26]), erectile dysfunction (IIEF [29]), various lower urinary tract symptoms (FLUTS [27]) or patients surgically treated for rectal cancer (Stoma, Pain and Female Sexuality Scores [24, 25, 28]). The Rectal Cancer Pain Score has recently been validated for use in patients surgically treated for colon cancer [47]. Only one study has assessed urinary and sexual function in 25 men following diverticulitis surgery, using the IPSS for urinary function and the IIEF‐5 for sexual function [48]. No PROMs have been applied to assess stoma dysfunction, chronic pain or female urinary and sexual dysfunction in diverticulosis. Consequently, no validated PROMs exist for this cohort. QoL has been extensively studied in Danish colorectal cancer patients [49, 50, 51], and the questionnaire content in the DIVIPACT study aligns with cross‐sectional screening for late sequelae following colorectal cancer surgery in the Northern and Central Denmark Regions [52].

### Strengths and limitations

The DIVIPACT cohort represents one of the largest cohorts used to examine the burden of diverticulosis. Key strengths include the large cohort and high response rate, which enhance the generalizability of findings and minimize selection bias. Furthermore, the ability to link self‐reported data with clinical registry information facilitates a comprehensive assessment of hospital contacts and surgical interventions as well as imaging, laboratory and pathological findings. This integration enables adjustment for confounding factors that have not often been accounted for in previous research.

However, the reliance on self‐reported data introduces potential recall bias, even though most questions focused on a relatively short recall period (2–4 weeks). Missing data presented a further limitation, addressed by conducting analyses on available responses, with proportions calculated for each specific item. Additionally, the cross‐sectional design precludes causal inference, offering only a snapshot of the burden of diverticulosis. Accordingly, a matched control group without diverticulosis would strengthen the inference that reported symptoms are attributable to diverticulosis rather than competing conditions.

## CONCLUSIONS

Diverticulosis is one of the most prevalent gastrointestinal conditions, and its burden is expected to grow as life expectancy increases. Many challenges in managing this condition stem from limited insights into its functional and psychosocial impact. The DIVIPACT cohort was designed to address these gaps.

The DIVIPACT cohort provides an outstanding database for conducting clinical research in the future. This publication introduces the cohort and its methodology, highlighting overall key findings on the impact of diverticulosis on daily life. Future research will investigate the validated PROMs and registry data and provide deeper insight into clinical outcomes, healthcare utilization and disease progression. By fostering a better understanding of the complex dimensions of the burden of diverticulosis, this work has the potential to provide the foundation for more tailored and effective patient‐centred management strategies in the future.

## AUTHOR CONTRIBUTIONS

**Helene R. Dalby:** Conceptualization; investigation; funding acquisition; writing – original draft; methodology; validation; visualization; software; data curation; formal analysis; project administration. **Rune Erichsen:** Conceptualization; writing – review and editing; supervision. **Kåre A. Gotschalck:** Conceptualization; writing – review and editing; supervision. **Katrine J. Emmertsen:** Conceptualization; funding acquisition; writing – review and editing; supervision; methodology; project administration.

## FUNDING INFORMATION

Funded by The Novo Nordisk Foundation (grant ref. no. NNF21OC0071125).

## CONFLICT OF INTEREST STATEMENT

The authors report no conflicts of interest in this work.

## ETHICS APPROVAL STATEMENT

The study was registered at the Danish Data Protection Agency in the Central Denmark Region (record no. 1‐16‐02‐61‐22), and the disclosure of information was approved by the Central Denmark Region (record no. 1‐45‐70‐19‐22).

## PATIENT CONSENT STATEMENT

Written informed consent was obtained from all participants.

## Supporting information


**Table S1.** Supporting information.

## Data Availability

The data that support the findings of this study are available on request from the corresponding author. The data are not publicly available due to privacy and ethical restrictions.
