# Supplementary material for: The DIVIPACT cohort profile: Evaluating the impact of colonic diverticulosis on daily life
Source: Colorectal Dis. 2025 Jun 27;27(7):e70155. doi: 10.1111/codi.70155 (PMC12205114; doi:10.1111/codi.70155)
Supplement: Supplementary file 1 — Table S1. Supporting information. [file CODI-27-0-s001.pdf]

Table S1: Content of the questionnaire

**Topic**	**Patient-reported outcome measures /** *ad hoc single items*	**Items**
Ad hoc		
Health factors	Height and weight	2
	Comorbidities (10 categories)	1
	Self-rated performance status	1
	Smoking status	1
	Alcohol consumption	1
	Physical activity	1
	Diet	11
Diverticulosis history	*Did you know you have diverticulosis?*	1
	How many days during the past 4 weeks did your diverticulosis prevent you from performing your daily activities such as work?	1
	Flare-ups treated at general practitioner with or without antibiotics	2
	Surgery due to diverticular disease (with or without stoma)	1
	How have you experienced the availability of counselling or advice regarding living with colonic diverticula?	1
Bowel habits	Rectal bleeding	3
	Medications (bulking agents, anti-diarrheal, laxatives)	3
**Patient-reported outcome measures**		
Bowel function	Bristol stool scale chart	1
	Overall, how much does your bowel function affect your quality of life?	
Stoma function	The Colostomy Impact Score	7
Disease-specific quality of life	Diverticulitis quality of life (DV-QoL)	17
Generic quality of life	European Quality of Life-5 Dimensions (EQ-5D-5L)	5
	European Quality of Life-5 visual analogue scale (VAS)	1
	Overall, how do you rate your quality of life over the past 4 weeks?	1
Pain	*Do you experience pain in the abdomen, lower abdomen, or pelvic region?*	1
	Modified Rectal Cancer Pain Score	6
Urinary function	ICIQ Female Lower Urinary Tract Symptoms (FLUTS)	12
	ICIQ Male Lower Urinary Tract Symptoms (MLUTS)	13
Sexual function	Modified Rectal Cancer Female Sexuality score	8
	International Index for Erectile Dysfunction (IIEF)	16
